# Insights into the dosimetric and geometric characteristics of stereotactic radiosurgery for multiple brain metastases: A systematic review

**DOI:** 10.1371/journal.pone.0307088

**Published:** 2024-08-09

**Authors:** Godfrey Mukwada, Crispen Chamunyonga, Pejman Rowshanfarzad, Suki Gill, Martin A. Ebert

**Affiliations:** 1 Department of Radiation Oncology, Sir Charles Gairdner Hospital, Hospital Ave, Nedlands, Western Australia, Australia; 2 School of Physics, Mathematics and Computing, University of Western Australia, Crawley, Western Australia, Australia; 3 School of Clinical Sciences, Discipline of Radiation Therapy, Queensland University of Technology, Brisbane, Queensland, Australia; 4 Centre for Advanced Technologies in Cancer Research (CATCR), Perth, Western Australia, Australia; 5 School of Medicine and Population Health, University of Wisconsin, Madison, Wisconsin, United States of America; The First Hospital of Jilin University, CHINA

## Abstract

**Background:**

GammaKnife (GK) and CyberKnife (CK) have been the mainstay stereotactic radiosurgery (SRS) solution for multiple brain metastases (MBM) for several years. Recent technological advancement has seen an increase in single-isocentre C-arm linac-based SRS. This systematic review focuses on dosimetric and geometric insights into contemporary MBM SRS and thereby establish if linac-based SRS has matured to match the mainstay SRS delivery systems.

**Methods:**

The PubMed, Web of Science and Scopus databases were interrogated which yielded 891 relevant articles that narrowed to 20 articles after removing duplicates and applying the inclusion and exclusion criteria. Primary studies which reported the use of SRS for treatment of MBM SRS and reported the technical aspects including dosimetry were included. The review was limited to English language publications from January 2015 to August 2023. Only full-length papers were included in the final analysis. Opinion papers, commentary pieces, letters to the editor, abstracts, conference proceedings and editorials were excluded. The Preferred Reporting Items for Systematic Reviews and Meta-Analyses guidelines were followed. The reporting of conformity indices (CI) and gradient indices, V12Gy, monitor units and the impact of translational and rotational shifts were extracted and analysed.

**Results:**

The single-isocentre technique for MBM dominated recent SRS studies and the most studied delivery platforms were Varian. The C-arm linac-based SRS plan quality and normal brain tissue sparing was comparable to GK and CK and in some cases better. The most used nominal beam energy was 6FFF, and optimised couch and collimator angles could reduce mean normal brain dose by 11.3%. Reduction in volume of the healthy brain receiving a certain dose was dependent on the number and size of the metastases and the relative geometric location. GK and CK required 4.5–8.4 times treatment time compared with linac-based SRS. Rotational shifts caused larger changes in CI in C-arm linac-based single-isocentre SRS.

**Conclusion:**

C-arm linac-based SRS produced comparable MBM plan quality and the delivery is notably shorter compared to GK and CK SRS.

## Introduction

Radiation therapy has been a mainstay modality for the management of many types of cancer. However, despite efforts to eradicate the disease, approximately 20–40% of cancer patients develop brain metastases [1–10]. Depending on the number of brain metastases, available technical resources and professional competency, a choice of whole brain radiotherapy (WBRT), stereotactic radiosurgery (SRS), stereotactic radiation therapy (SRT) or a combination of WBRT, and SRS can be administered. A large body of evidence demonstrates that both SRS (high dose in a single fraction) and SRT (high dose delivered in 1–5 fractions) result in less neurocognitive decline compared with WBRT [1, 6, 11, 12]. Due to their neurocognitive sparing, and positive impact on quality of life, SRS or SRT is now the standard of care for oligometastases and is increasingly preferred for all brain metastases [12, 13]. In a case-matched study comparing the results of 2–9 brain metastases against more than 10 brain metastases, Yamamoto *et al*., [14] demonstrated that SRS for more than 10 metastases was not unfavourable. This study along with several others [15–19] has resulted in increased clinical demand for SRS for multiple brain metastases (MBM).

SRS and SRT treatment planning and delivery systems for MBM are varied and include Gamma Knife (GK), CyberKnife (CK), Tomotherapy, C-arm linear accelerator (linac) based systems and the recently developed Zap-X radiosurgery system. GK was the first SRS-dedicated solution, and its current design makes use of 192 Cobalt-60 sources that produce precisely focused radiation beams at isocentre through fixed collimators. The CK utilises a linear accelerator that is mounted on a robotic arm guided by real-time imaging. CK SRS delivery is through one of the following beam collimations; cones, Iris (hexagon shaped aperture) and InCise multileaf collimators (MLCs) [20]. Tomotherapy is a general-purpose radiation therapy system that delivers an MLC-modulated fan radiation beam from a bore-mounted rotating source coupled with helical couch movement [21, 22]. C-arm linac-based SRS has several variations that are linked to the individual vendor’s ingenuity or a combination with other vendors’ solution such as Brain Elements Treatment Planning solution (BETPS) also referred to as Brainlab Multiple Mets Elements (Brainlab MME) (BrainLab Inc., Munich, Germany) delivered via a Varian linear accelerator. Delivery is via cones or small-width MLCs (= <5mm at isocentre) through dynamic arc therapy (DCAT) or intensity modulated beams coupled with advanced imaging. Zap-X is a self-shield 3 MV linear accelerator mounted on a gimbal system that is capable of delivering non-coplanar beams and has inbuilt kilovoltage imaging capability allowing real time imaging [23]. Of these planning and delivery solutions, GK and CK are dedicated SRS solutions and are generally regarded as the gold standard in the treatment of MBM [24–28]. Although Zap-X is a dedicated SRS system, its first clinical use was in 2019 so published clinical experience is limited, and is evolving as a technique as demonstrated by frequent software updates [29–31].

Traditional C-arm linac-based SRS approaches for MBM treatment involve multiple-isocentre planning and delivery is time consuming as the metastases are treated one by one, with the patient shifted and isocentre verified before each lesion is treated. Technological advancements in treatment planning systems, high-definition MLCs, frameless masks (immobilisation), automated 6 degrees of freedom (6DoF) couch corrections and improved imaging capabilities have transformed MBM SRS. These recent developments favour a single-isocentre technique [32] with studies demonstrating non-inferiority to the traditional multiple-isocentre technique, except for reduced dose coverage due to rotation shifts which can be mitigated [13, 33, 34]. Although BETPS pioneered single isocentre MBM delivered via conformal arcs, VMAT based MBM SRS is flourishing more recently due to availability.

A systematic review by Rozati *et al*.,[35] on overall survival following SRS for 10 or more brain metastases showed GK to have less necrosis (12.2%) compared to C-arm linac based SRS (35%). Several overall survival studies conducted by De La Lena *et al*.,[36] on patients with MBM treated with CK between 2011 and 2017 reported no late complications such as necrosis, with local control exceeding 90% at 1 year.

The variation in radio-necrosis rates is likely attributed to the larger planning target volume (PTV) margins typically used in C-arm linac-based SRS compared to GK and CK. Advances in imaging, immobilisation and the development of fine isocentre SRS machines, is contributing to the reduction in PTV margins for C-arm linac based SRS [6, 13, 32] potentially reducing toxicity and overall survival across different SRS technologies. Furthermore, there are many studies on overall survival for MBM GK and CK SRS deliveries [14, 16, 35–41] and there is a need for such studies for C-arm linac based for a fair comparison [42].

Most SRS techniques are frameless and require thermoplastic masks for immobilisation. Initial translational and rotational setup errors are corrected through couch translations and 6DoF couch rotations respectively. Despite accurate initial patient positioning, several studies have demonstrated intra-fraction motion in SRS [43–47]. Dosimetry for the single-isocentre technique can be adversely impacted by the resulting positioning errors [47, 48]. Nakano *et al*., [49] recommend a maximum PTV to isocentre distance of 5.5cm for a 1.5mm diameter target and 0.5° rotation, beyond which loss of dose coverage is clinically significant. Prentou *et al*., [48] recommend a maximum distance of 4cm from isocentre.

The quality of SRS treatment plans is generally evaluated using the following dosimetric parameters; Paddick conformity index (CI_p_), RTOG conformity index (CI_RTOG_), Paddick gradient index (GI_p_), V12Gy, number of MUs, among others. CI_p_, CI_RTOG_ and GI_p_ are defined as per Clark *et al*., [50] and Paddick [50, 51] as shown in Eqs 1–3.

CIp=(TVPV)2TV×PV (1)

CIRTOG=PVTV (2)

GIp=PV50%PV (3)

Here, TV is the target volume, TV_PV_ is the target volume covered by the prescription isodose, PV is the volume of the prescription isodose, PV_50%_ is the volume of the isodose line at 50% of the prescription.

The modified Paddick conformity index (nCI) is also reported, being essentially the inverse of the CI_p_ [52, 53]. Understanding the current use of these indices in the context of recent technical advances is of paramount importance for standardising their utilisation in clinical departments. Homogeneity indices (HI) are not directly relevant to SRS as the GTV or PTV dose tends to be inherently inhomogeneous. The limitations of the current plan metrics are acknowledged and a novel efficiency index that depends on the integral dose has been proposed [54].

Contemporary SRS treatments are prescribed to the isodose surface that covers the optimal percentage of the PTV while maintaining an optimally reduced dose to organs at risk (OAR) [55]. The prescription isodose surface depends on the SRS technique; for example, GK prescriptions are usually to the 50% isodose surface, CK prescriptions are made at around the 70–85% isodose surface while the C-arm linac-based approach uses approximately the 75–90% isodose surface covering the optimal PTV. Several authors demonstrated that the prescription isodose diameter influences the GI [56].

Relative affordability, fast delivery and comparable treatment outcomes have fuelled the increase in C-arm linac-based SRS relative to GK and CK [8, 57]. Volumetric arc therapy (VMAT) or dynamic conformal arc therapy (DCA) approaches have enabled innovation in single-isocentre SRS. This review aims to summarise and evaluate plan quality indices and the impact of geometric shifts on dose coverage in MBM SRS. Furthermore, the radiation dose to the brain healthy tissue (Brain minus PTV) will be evaluated. Ultimately, the review seeks to establish whether the contemporary MBM SRS techniques compare with the mainstay MBM SRS techniques in terms of achievable quality and deliverability.

## Methods

### Search and screening

**Fig 1:**
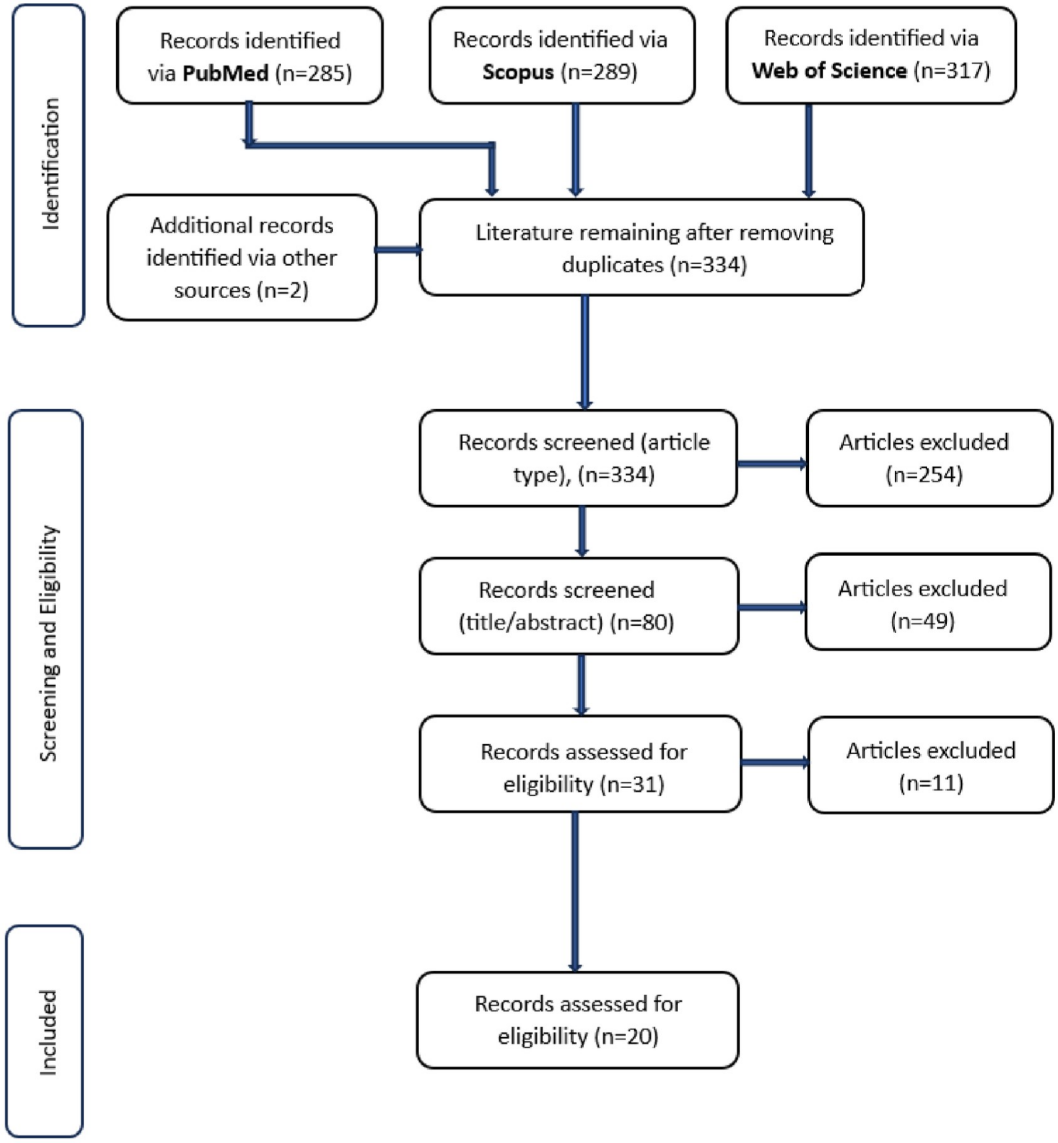
PRISMA flow chart for literature search strategy [58].

A systematic review of the literature was performed using the Preferred Reporting Items for Systematic Reviews and Meta-Analyses (PRISMA) guidelines [58]. The associated flow chart is shown in Fig 1. Peer-reviewed articles were sourced from PubMed, SCOPUS, and Web of Science databases, and manual search of the principal relevant journals and the last search was on 30 August 2023. According to Haddaway *et al*., [59] searching 5–10 databases offers no significant advantage over searching 2–3 relevant databases. The selected databases for this review are highly reputable scientific resources pertinent to the study, mitigating selection bias. To reduce publication bias [59, 60], additional searches were undertaken manually via Google Search and Google Scholar. Relevant search terms and the records identified through primary and secondary search are shown in Table 1. Relevant syntax operators including Boolean operators, additional filters and limiters were used. The articles retrieved were exported to EndNote version 20. To avoid bias, two authors independently assessed the remaining articles for eligibility. The final articles were double-checked for eligibility by two separate authors. Furthermore, the Critical Appraisal Skills Programme (CASP) checklist forms were completed (S1 Checklist). CASP comprises 10 specific questions suitable for assessing potential bias in articles for systematic reviews.

**Table 1:** Search strategy for MBM SRS.

Search type	Search terms	PubMed	Scopus	Web of Science
**Primary**	"Multiple brain metastases" AND "stereotactic radiosurgery" OR "multiple brain targets" AND "stereotactic radiosurgery" OR "multiple brain metastases" AND "stereotactic radiation therapy" OR "multiple brain metastases" AND "stereotactic radiotherapy".	227	225	281
**Secondary**	"CyberKnife" AND "multiple brain metastases" OR "GammaKnife" AND "multiple brain metastases" OR "Linac stereotactic radiosurgery" AND "multiple brain metastases"	58	64	36

### Inclusion and exclusion criteria

Primary studies which reported the use of SRS for treatment of MBM SRS including SRT and reported the technical aspects including dosimetry were evaluated based on the selection criteria. To eliminate screening bias, the inclusion and exclusion criteria were determined beforehand. In addition, bias towards particular research groups or regions were addressed by screening the abstracts and manuscripts based on content and not authors or participating centres. The review was limited to English language publications from January 2015 to August 2023. Only full-length papers were included in the final analysis. Opinion papers, commentary pieces, letters to the editor, abstracts, conference proceedings and editorials were excluded. Primary studies that had a maximum of 1 metastasis were excluded. Some relevant records that did not meet the inclusion criteria are included in the discussion.

### Descriptive analysis

Treatment technical aspects including the treatment modalities and the quality indices were extracted manually from the articles under review to a Microsoft excel spreadsheet and are shown in Table 2. Also, reported information on geometric shifts and their impact on dose coverage were extracted. The extracted data were insufficient for rigorous statistical analysis, so was not used. Instead, graphs and plots were employed for easier data visualization and interpretation.

**Table 2:** Summary of articles under review. CI_p_>1 is nCI. SI-, MI-, DI-, RSI-, and C- stands for single-isocentre, multiple isocentre, dual isocentre, restricted isocentre, and conventional, respectively. A dash indicates missing information in the respective study.

Reference	Year	Techniques	TPS	Algorithm, grid size (mm)	PTV margin (mm)	Delivery System	Energy	no. of tumours per case (total), (patients)	No. of ISOs	GI_p_	CI_p_ (CI_RTOG_)	HI_p_ (HI_RTOG_)	V12Gy	MU
Chang *et al*. [68]	2018	MI-DCA vs RSI-DCA (MME)	Iplan MME	Pencil beam, 2.0	2	-	-	2–6 (38), (10)	M+	-	(1.4, 1.25)	-	2.2, 2	-
Chea *et al*. [25]	2021	GK vs DCA (MME)	Gamma Plan v.11, MME v.2	Pencil beam	0	GK, Varian TrueBeam STx, 2.5mm	Co60, 6FFF	2–9 (95), (20)	1, M+	4.09, 3.22	0.57, 0.532	-	-	-
De Camargo *et al*. [66]	2021	VMAT	Eclipse v.13.6,	AAA, 1.25	1	Varian TrueBeam STx, 2.5mm	-	4–21 (168), (17)	1, M+	-	(+1.2)	13.53 (12.91)	12.91	-
El Shafie *et al*. [52]	2020	CK vs SI-VMAT	Multiplan v.5.3, RayStation VB8	Ray tracing, collapsed cone.	1 (CK), 3(VMAT)	CK M6, Versa HD, 5mm	6FFF,	5–10 (), (20)	1, M+	3.1, 5.0	1.2, 1.6 (1.2, 1.5)	-	6.5, 37	130, 13.7
Homfaier *et al*. [63]	2019	DCAT vs SI-VMAT	MBSRS v.1.1, Monaco v.5.11	Pencil beam (1.5mm), XVMC (2mm)	1 (DCA), 2(VMAT)	-	-	2–6 (66)	1	5.99, 7.17	0.75, 0.73	-	2.1, 3.1	4569, 5840
Hossain *et al*. [69]	2016	GK vs VMAT	Gamma Plan v.11, Eclipse v.11	2.5 grid	-	GK, Varian TrueBeam STx, 2.5mm	Co60, 6FFF	1–12 (5)	M+	-	-	-	1.9, 4.0	-
Kadoya *et al*. [26]	2019	CK vs HyperArc	Multiplan, Eclipse v.15.5	Ray Tracing, Acuros, 1.0	-	CK M6, Varian TrueBeam STx, 2.5mm	6FFF	3–5 (45)	1, M+	3.94, 5.31	0.60, 0.87	1.72, 1.44	5.26, 4.02	-
Laoui *et al*. [70]	2023	SI-VMAT 10 vs 6FFF	Eclipse,	AAA, 1.0	-	Varian TrueBeam STx, 2.5mm	10FFF, 6FFF	1–9 (93)	1	4.70, 4.56	(1.11, 1.10)	-	-	-
Liu *et al*. [27]	2016	GK vs SI-VMAT	Gamma Plan v.10.1, Eclipse v.11	AAA, 1.0	-	GK, Varian TrueBeam STx, 2.5mm	Co60, 6FFF	3–4 (19)	1, M+	3.65, 4.77	(1.50, 1.19)	-	3.06, 2.73	72, 6.4
Ohira *et al*. [67]	2018	HyperArc vs MI-VMAT	Eclipse v.13.7,	AAA	CTV = GTV+2, PTV = CTV+1	Varian TrueBeam STx, 2.5mm	6FFF	1–4 (41)	1 & 2+	3.06, 3.91	0.93, 0.90	1.41, 1.24	7.9, 11.3	8186, 6758
Palmiero *et al*. [57]	2020	SI-VMAT vs DI-VMAT	Eclipse v.15.6,	AAA, 1.25	1	Varian TrueBeam, 5mm	10FFF	5–16 (64)	1 & 2	-	0.98, 0.97	-	1.19	12497, 10608
Pokhrel *et al*. [71]	2021	HyperArc vs DCA-VMAT	Eclipse v.15.6	AAA, 1.25	1	Varian TrueBeam, 5mm	10FFF	2–8 (35)	1	-	(1.23, 1.25)	-	55.3, 39.7	12498, 5742
Prentou *et al*. [48]	2020	SI-VMAT	Monaco v.5.10	XVMC, 1.00	2	Agility, 5mm	6FFF	3–4 (36)	1 & 2	5.34, 4.72	0.735, 0.76	-	-	
Priyadarsini *et al*. [72]	2022	SI-VMAT	Eclipse v.15.6	Acuros, 1.25	-	Varian TrueBeam, 5mm	6FFF	5–10 (45)	1	4.74	0.91 (1.02)	(+1.49)	-	-
Ruggieri *et al*. [33]	2018	HyperArc vs MI-VMAT	Eclipse v.15.6	AAA, 1.00	2	Varian TrueBeam, 5mm	-	2–10 (99)	1 & M+	4.41, 6.08	0.96, 0.87	-	23.6, 42.2	4313, 15150
Tsui *et al*. [46]	2022	SI-VMAT vs MI-VMAT	Eclipse v.15.6	AAA, 2.00	1–2	Linac, m3	6FF	2–3 (46)	1 & M	-	0.83, 0.84	-	-	-
Turkkan *et al*. [64]	2022	VMAT vs DCA	Monaco v.5.10	XVMC, 2.00, 1%	2	Versa HD,	6FFF	2–10 (84)	1	5.86, 8.01	(1.21, 1.78)	(1.38, 1.17)	-	3097.44, 1479.09
Velten *et al*. [53]	2021	DCA vs SI-VMAT	Iplan MME, Eclipse v.15.6	Pencil Beam 1.0, AAA, 1.00	1	Varian TrueBeam STx, 2.5mm	6FFF	2–18 (103)	1	-	1.45, 1.38	-	7.6, 17.9	9049, 10910
Wu *et al*. [65]	2016	VMAT opt. vs non-opt.	Eclipse v.13.5	AAA, 1.00	2	-	6FFF	3–5 (53)	1	5.26, 5.24	1.24, 1.25	1.16, 1.16	3.06	-
Zhang *et al*. [28]	2017	CK vs SI-VMAT, C-VMAT, IMRT	Eclipse v.13.6, Multiplan	AAA, 1.25, Ray Tracing	2	Varian TrueBeam, 2.5mm, CK Iris	6FFF	2–5 (48)	1	3.49, 4.21, 4.87, 5.36	(0.86, 0.87, 0.86, 0.85)	-	94.97, 124.69, 165.98, 168.56	28733.59, 3105.2, 2997.27, 4128.40

### Dosimetric analysis

It was assumed that the quality indices reported in the studies are comparable within the confines of minor differences in treatment planning systems (TPSs) index calculations, with calculation grid sizes assumed small enough for consistency [61]. The extracted mean CI_p_, nCI, CI_RTOG_, GI_p_ and normal brain tissue irradiated by a dose of at least 12 Gy (V12Gy) were presented in chart format for easy visualisation and interpretation. Among other factors such as the beam quality, V12Gy is also influenced by the tumour size and total tumour volume (all tumours) [25, 57, 62] and hence the mean total volumes we also extracted. There were large variations in treatment times (including set-up) across techniques. For easy analysis and interpretation, MU ratios were calculated by dividing the MUs for an established technique by those for the comparator technique in the respective article or study. For example, in the study by Homfaier *et al*., [63] in Table 2, the DCA technique is considered established and is being compared with the SI-VMAT technique, and thus MUs for the DCA provide the normalisation. The range of MUs were also quoted for context.

### Geometric shift analysis

Articles simulating translational and rotational shifts were reviewed, and the mean change in conformity index due to these shifts was analysed. Influencing parameters were considered, such as the distance between the centres of mass of individual targets.

## Results

### Descriptive analysis

The search strategy identified a total of 891 records which were narrowed down to 20 articles after applying the inclusion and exclusion criteria. Three studies included a comparison with GK, and another three studies made a comparison with CK. Among linac-based SRS studies there were 19 VMAT, 6 DCA, and only 1 intensity modulated radiation therapy (IMRT) delivery. These machines were coupled with different TPSs namely, 4 Iplan MME, 14 Eclipse, 3 Multiplan (CK), 3 Gamma plan (GK), 3 Monaco and 1 RayStation. Sixteen out of the 20 studies considered more than 4 metastases and the highest number of metastases per patient was 21. Some authors reported GTV total volume while others reported PTV total volume. The GTV total volume ranged from 0.02–10.5 (1.2–11.1) cc and PTV total volume ranged from 0.1–15.6 (0.5–38.6) cc and two outliers PTV total of 70.6 and 74.3 cc. PTV margin ranged from 0–3 mm. Where the PTV margin was not explicitly stated, the authors assumed the same PTV margin was used for all techniques under comparison. Nine studies reported MLCs with 2.5 mm width at machine isocentre, 5 with 5mm width, one reported use of an m3 micro-MLC (Brainlab Inc., Munich, Germany), and one use of a Versa HD MLC. Nominal energies included 16 x 6FFF, 1 x 6FF, 3 x 10FFF, and 3 x 192 Co-60 (GK). Eighteen of the 20 studies reported a single-isocentre technique study by itself or in comparison to a multiple-isocentre technique. Eclipse AAA was the most used dose calculation algorithm, and the most frequently used calculation grid sizes were in the range 1–1.25 mm.

### Dosimetric analysis

**Fig 2:**
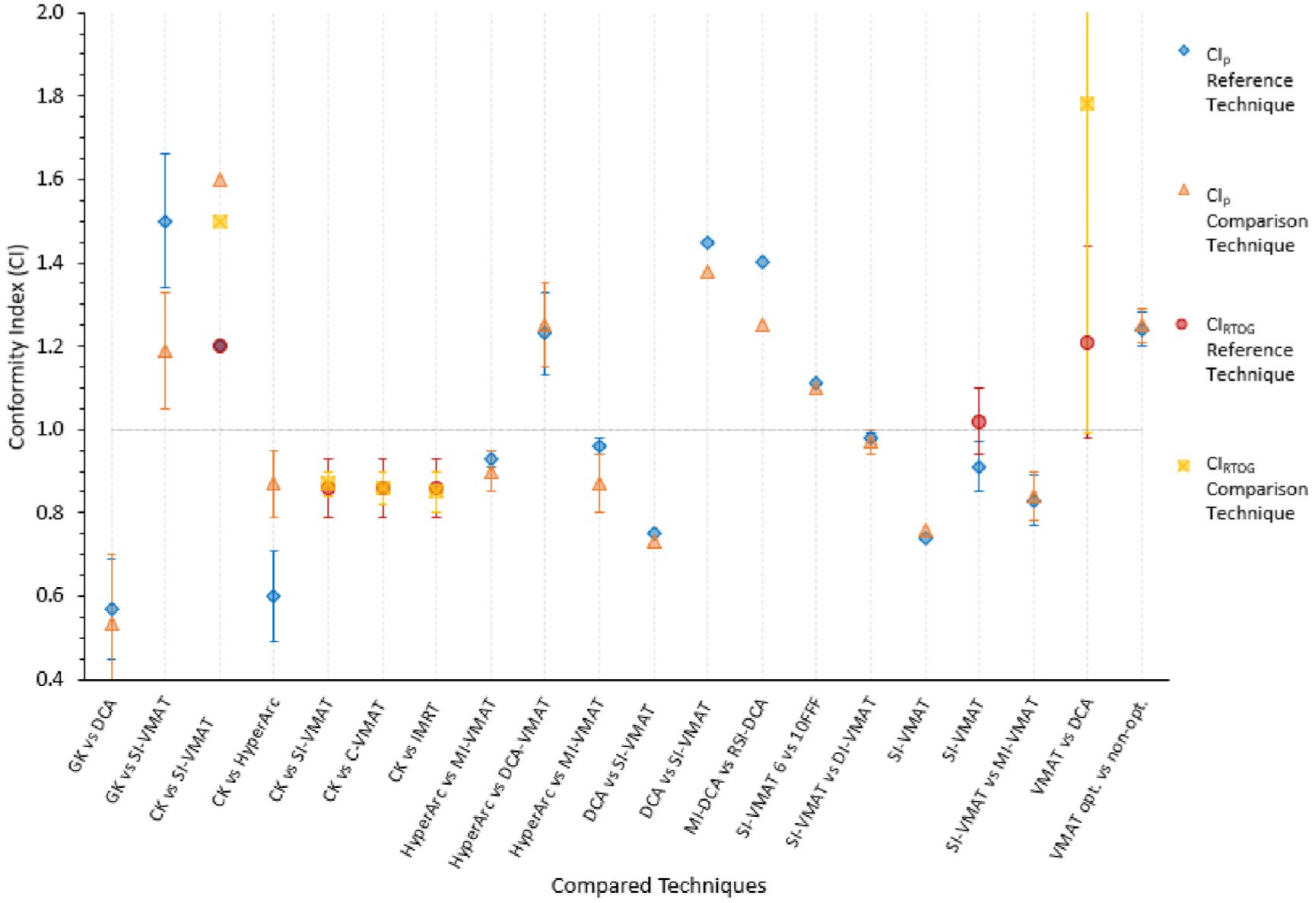
Mean CI_p_, nCI & CI_RTOG_ compared per study. For each column the first written technique is the reference/established one. For example, for MI-DCA vs RSI-DCA, MI-DCA is the reference. Some of the error bars were clipped to allow better visualisation. CI with no error bars had no standard deviation (stdev) in the respective studies. To simplify the figure, CI_p_ above 1.0 refers to nCI.

**Fig 3:**
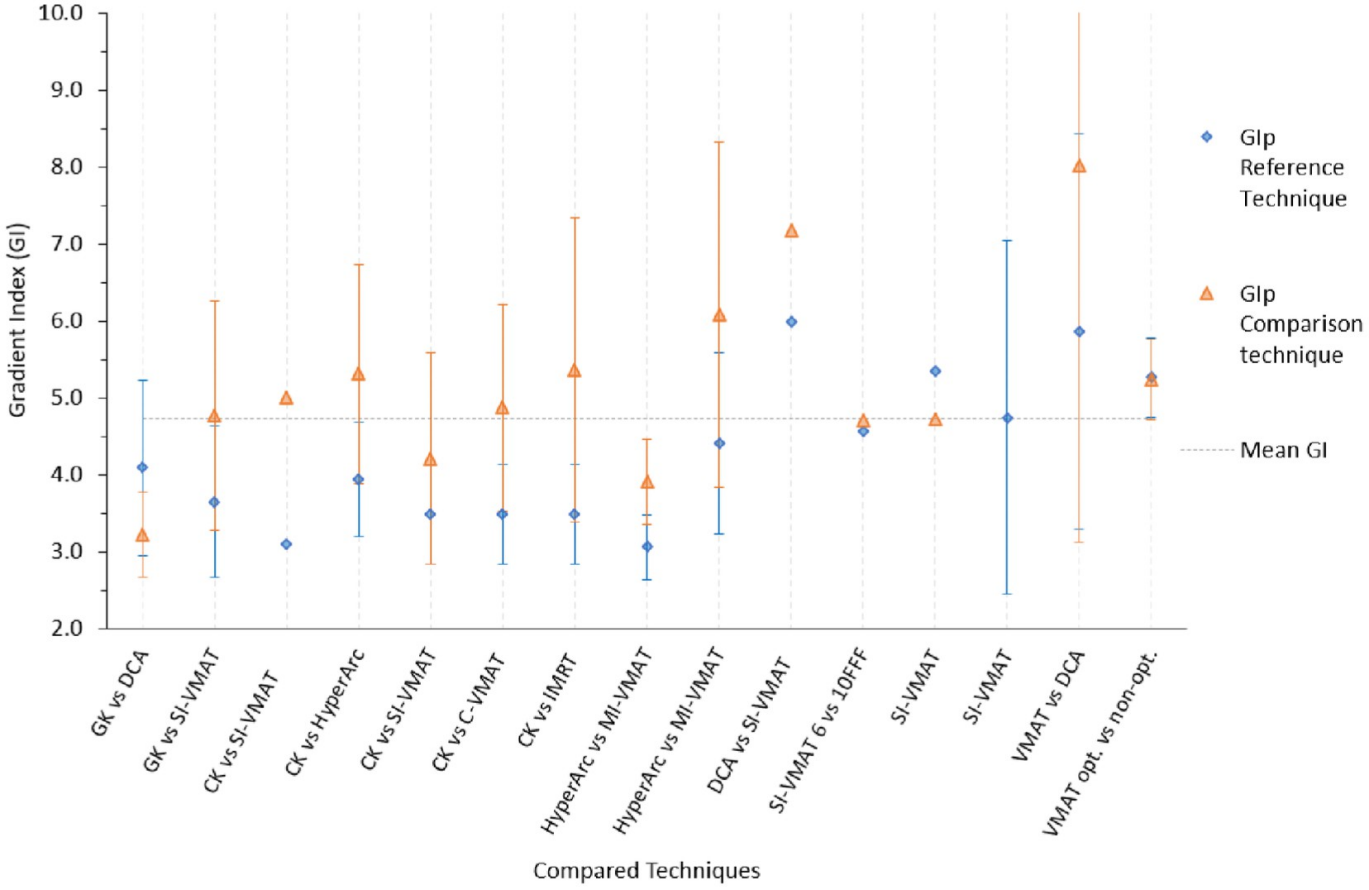
Mean GI_p_ compared per study. For each column the first written technique is the reference/established one. For example, for MI-DCA vs RSI-DCA, MI-DCA is the reference. GI with no error bars had no stdev in the respective studies.

**Fig 4:**
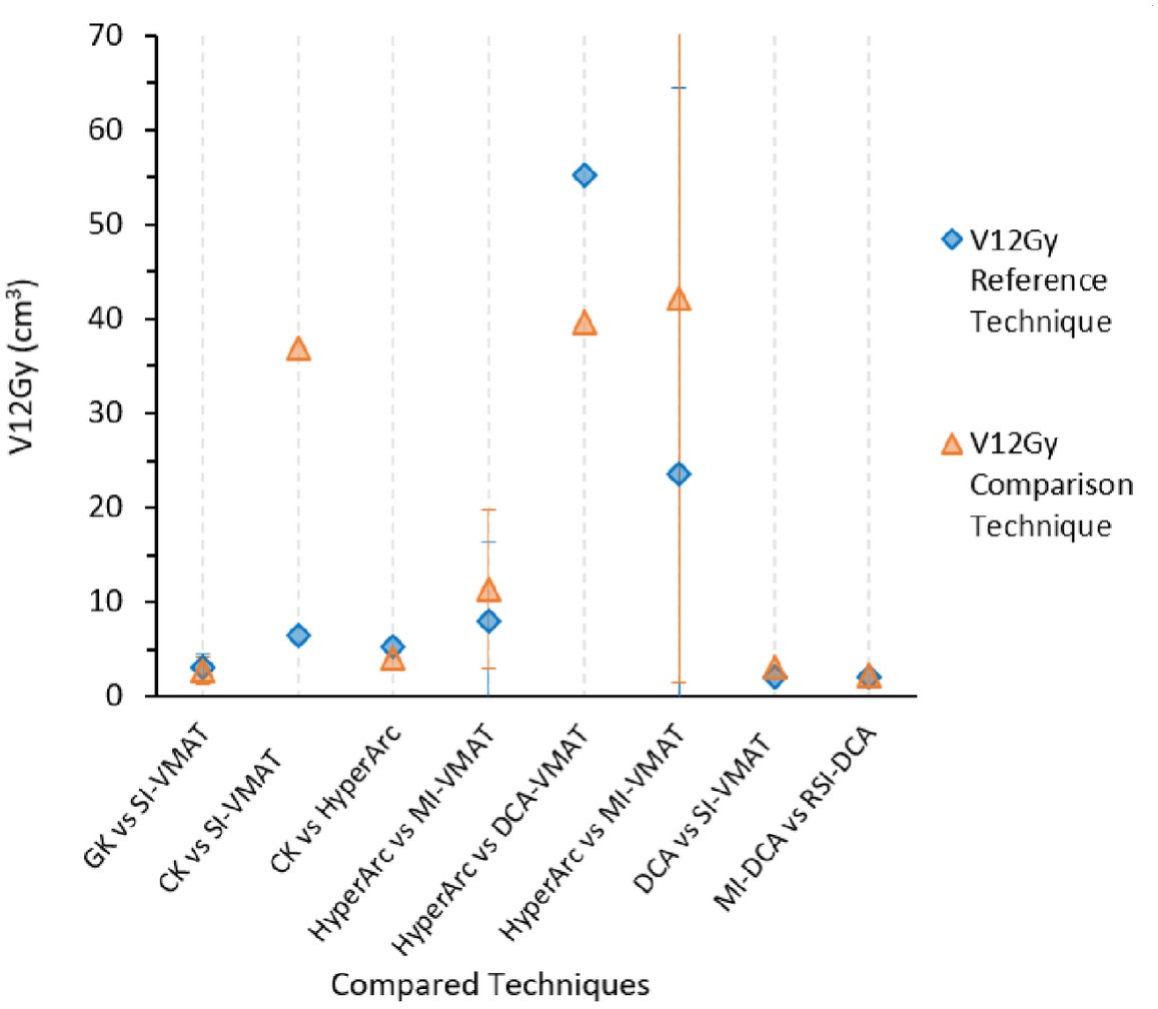
Mean V12Gy for compared per study. For each column the first written technique is the reference/established. For example, for MI-DCA vs RSI-DCA, MI-DCA is the reference.

Across the studies there were variations in the reporting of plan quality indices, tumour volumes and the dose to which the volume should be reported. Also, some studies reported mean values while others reported the median. In this review, the plans’ qualities were essentially evaluated per study and hence median was taken as mean to simplify data presentation. The variations of plan indices are shown in Figs 2–4. The ideal CI_p_, nCI, CI_RTOG_ should be 1. From Fig 2, the mean CI_p_ ranged from 0.532–0.97. The study with the largest CI_p_ difference was CK vs HyperArc, 0.27 (0.87–0.60) in favour of CK [26]. Mean nCI ranged from 1.10–1.60. The study with the largest nCI difference was CK vs SI-VMAT, 0.4 (1.60–1.20) in favour of SI-VMAT [52]. Mean CI_RTOG_ ranged 0.85–1.78. The study with the largest CI_RTOG_ difference was VMAT vs DCA, 0.57 (1.78–1.21) in favour of VMAT [64].

The ideal GI_p_ should be 3 or less [56]. The mean GI_p_ was 4.30. From Fig 3, the mean GI_p_ ranged from 3.06–8.01. The study with the largest GI_p_ difference was VMAT vs DCA, 2.15 (8.01–5.86) in favour of VMAT [64]. SI-VMAT 6 vs 10FFF and VMAT opt vs VMAT non-opt showed similar GI_p_ as shown in Fig 3. Overall, all studies showed the reference or established techniques to have better GI except GK vs DCA which favoured DCA [25].

**Fig 5:**
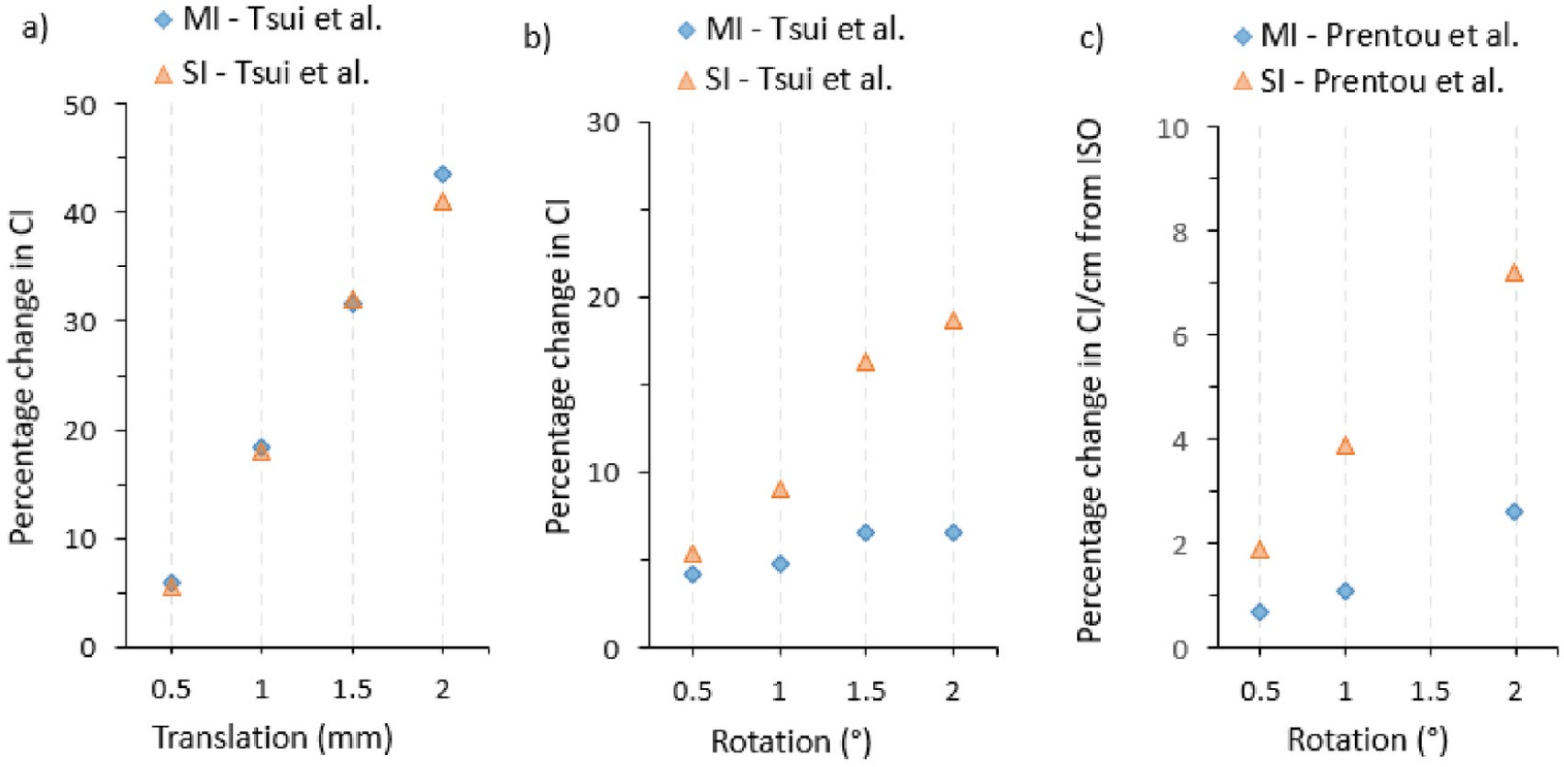
Percentage change in CI_p_ for MBM with respect to a) translation shifts, b) rational shifts and c) rotational shifts including distance from isocentre (iso).

Other studies reported V2Gy up to V16Gy, but only V12Gy was reported in Table 2 as many studies consider V12Gy > 10cm^3^ to be associated with radiation necrosis. From Fig 4, the mean V12Gy ranged from 2.00–55.30. The study with the largest V12Gy difference was CK vs SI-VMAT in favour of CK 30.5cm^3^ (37.0–6.50) [52]. In a study by Wu et al., [65] optimised in terms of couch and collimator angles reduced the V12Gy and mean normal brain dose by 3.1 ± 1.6% and 8.4 ± 2.9% respectively compared to an unoptimized VMAT technique. The reduction in the volume of the healthy brain receiving a certain dose was depended on the number and size of the metastases, the relative geometric location and amount of overlap. There was no uniform reporting of the PTV to isocentre distance impact on plan quality indices. In agreement, De Camargo et al., [66] reported and Tsui et al., [46] recommended a maximum of 3.6–3.7cm PTV to isocentre distance for ±2° rotational shift assuming a maximum allowable 0.2 change in CI. In Fig 5c. Prentou *et al*., showed a change in CI with distance from isocentre for 0.5°, 1.0° and 2.0° rational shift. In the worst rotational shift of ±2°, a 7.2% per cm change in CI was observed.

There was pronounced variation in treatment time or MUs across the SRS techniques. Liu et al., [27] found GK treatment times to be 4.5 times those of VMAT (72 mins for GK with new radiation sources and 16 mins for VMAT including setup and imaging). El Shafie *et al*., [52] reported median treatment time of 130 mins (CK) vs 13.7 mins (VMAT) including set up and imaging. Also, a study by Zhang *et al*., [28] found CK MUs to be 8.4 times compared to linac-based techniques. Overall, GK and CK require much more treatment time compared to linac-based SRS techniques. There is also significant variation in MUs for the linac-based SRS techniques as shown in Fig 5. HyperArc vs MI-VMAT showed the largest MU difference with the M-VMAT technique requiring 3.5 times MUs to compared to HyperArc [67].

### Analysis of geometric shifts

**Fig 6:**
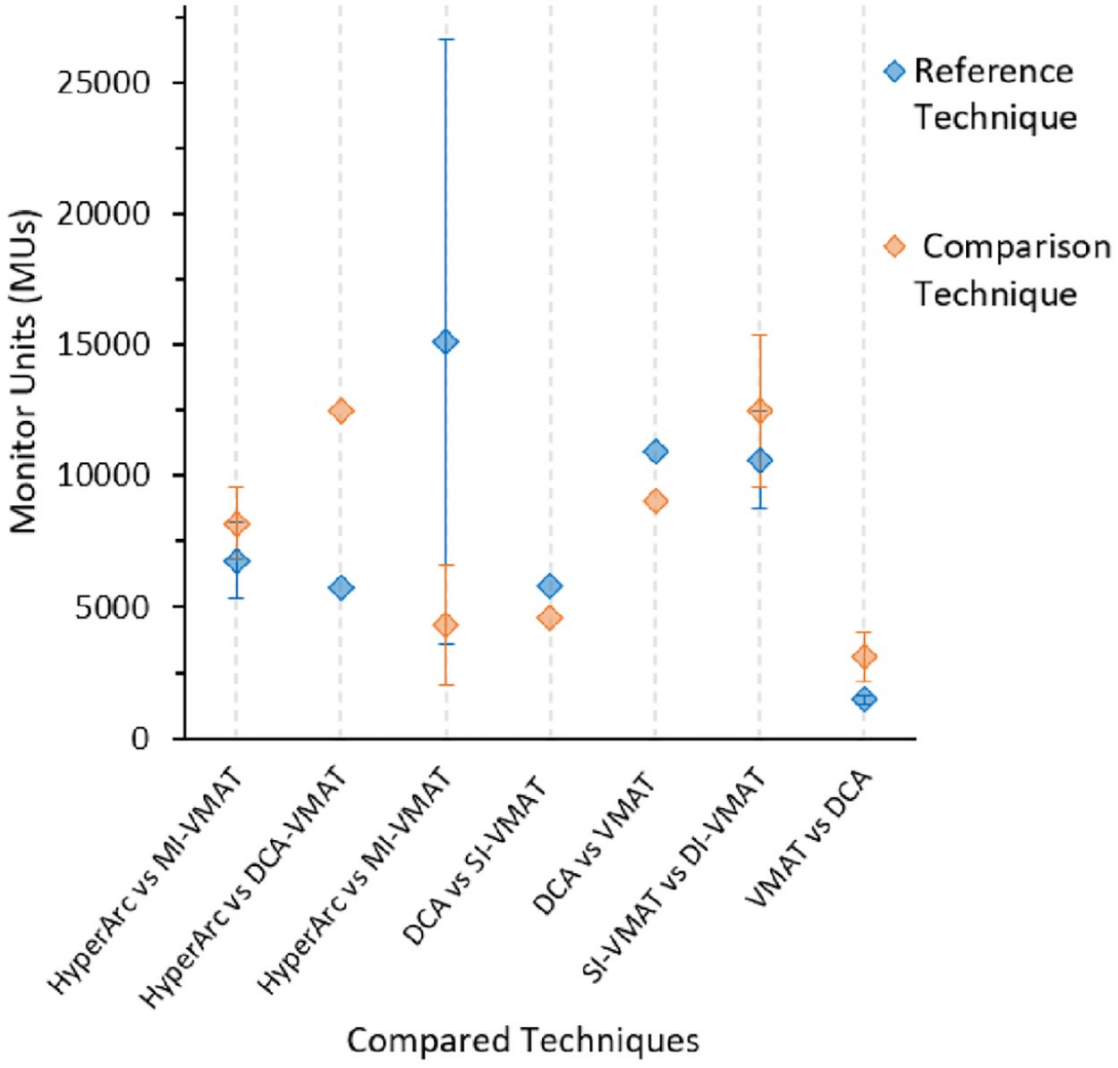
MU comparison per study. For each column the first written technique is the Reference. For example, for DCA vs SI-VMAT, DCA is reference.

The change in CI_p_ due to a translational shift of between ±2 mm was found to be similar for multiple and single-isocentre techniques as shown in Fig 5a. However, rotational shifts caused large changes in CI_p_ in single-isocentre compared to multiple-isocentre techniques as shown in Fig 6. Tsui *et al*., [46] showed that on average the conformity index decreases by a factor of 2.6 when rational shifts change between 0 and ±2°. Decreases in dose conformity of a factor of 3 were observed by Prentou *et al*., [48] for the same rational shifts.

## Discussion

### Descriptive analysis

There has been a growing interest in linac-based SRS over the past 8 years, especially because of the widespread availability of VMAT technology, 6DoF couch correction, and the increased uptake of large doses per fraction for body stereotactic radiotherapy for oligometastases and inoperable primary tumours. This may also be attributed to the greater accessibility of linacs in comparison to dedicated SRS delivery systems such as CK and GK. Rapid developments of linac associated capabilities and complimentary TPS capabilities seem to be the catalyst, transforming SRS delivery. Capitalising on VMAT capabilities and optimising treatment parameters such as couch and collimator angles has seen the rise of single-isocentre SRS technique. The availability of dedicated linac based SRS treatment planning solutions such as BrainLab MME BETPS and HyperArc have dominated SRS treatment techniques in recent years. The results show that conventional, optimised VMAT techniques and HyperArc hosted on Eclipse as the most studied SRS techniques in recent articles.

This review showed increased usage of FFF beams relative to the traditional FF beams. The FFF beam provides similar dosimetric coverage compared to FF beams but also offers sharper penumbra, lower dose spillage and peripheral dose, and increased dose rates [73–75]. Use of 6FFF was reported more than 10FFF—this could be due to energy availability, or preference due to the slightly sharper dose fall off exhibited by 6FFF, as reported by Laoui *et al*., [70]. MLC width plays a role in dose conformity and thinner MLCs are recommended especially for smaller tumours [6, 13, 52]. Nowadays, 5mm MLC width is a minimum standard on conventional C-arm linacs and the comparable quality of treatment plans achieved with thinner MLCs has contributed to the increase in linac-based SRS. This was demonstrated by a survey conducted across Australia and New Zealand that showed 60% of the centres offering MLC linac-based SRS were using 5mm MLC width [76]. The use of MLCs for CK SRS has also been reported [77] which makes the treatment delivery faster compared to cones.

In the studies reviewed, PTV margins ranged from 0 – 2mm except for one linac-based technique planned on RayStation TPS that used 3mm. This was a retrospective comparative study involving Multiplan with 1mm PTV margin for CK and then an additional 2mm for linac based SRS. GK and CK had 0 or 1mm PTV margin at most. The PTV margins are influenced by imaging, immobilisation, and availability of setup error correction methods such as via 6DoF. Such PTV margins are supported by the multicentre benchmarking planning studies conducted by Eaton *et al*., [24]. Vergalasova *et al*., [10] in their multi-institutional dosimetric SRS evaluation, started with 2mm PTV margin and are reducing to 1mm margin due to improved confidence in their technique. In a randomised trial by Kirkpatrick et al., [78] the prospective study found no difference in local control between 1mm and 3mm PTV margin groups. However, the study found increased incidences of radiation necrosis for the 3mm PTV margin. In conclusion the study recommends a 1mm PTV margin for linac-based SRS and this support by other studies [6, 10].

Although the individual GTV sizes are usually small (<1 cc), the GTV total volume and PTV total volume can be large. Several studies have indicated similar treatment outcomes for patients with less than 10 metastases compared to those with 10 or more [14, 41, 79, 80]. Yamamoto *et al*., [14] demonstrated that small tumour volume was a predictor of overall survival and not the number of metastases. Grandhi *et al*., [80] also showed that the treatment volume was a significant variable to survival and that the number of metastases was not a determinant of survival or local control. A case study of a patient with 37 metastases by Hyde *et al*., [81] cemented the above point because at 18 months the patient had 100% local control. Generally, as the number of metastases increases, the GTV total volume also increases. Unfortunately, there is no consensus on the limit of the GTV total volume suitable for MBM SRS and this is also demonstrated by the variation in the reviewed GTV and PTV total volume. The different GTV sizes and shapes and GTV total volume makes treatment planning complex. To reduce toxicity in treatments with large GTV total volumes, the strategies include fractionating SRS and reducing the total dose [13, 82].

More than 90% of the reviewed articles involved a single-isocentre SRS technique. Different C-arm linac-based single-isocentre SRS techniques have been compared against each other such as DCA vs HyperArc, Conventional VMAT vs HyperArc, and DCA vs Conventional VMAT. This trend reflects a significant interest and increased usage of the single-isocentre technique, likely due to its availability and affordability. At the core of radiation therapy is the precise and accurate delivery of radiation to the target while sparing healthy tissues. Comparative studies on SRS technology have shown a reduced gap between technologies in terms of delivery accuracy. Emerging SRS technologies, such as ZAP-X, are not only meeting the requirements for precise and accurate dose delivery but are also addressing other considerations, such as the elimination of the need for radiation-shielded bunkers [23, 31].

While it is standard in healthcare for informed consent to be provided before treatment [83], with such emerging SRS technologies, it is paramount to provide patients with adequate information about these alternatives that offer similar outcomes [84]. For example, a study by Mizuno et al., [16] compared SRS to WBRT as the initial treatment for 10–20 brain metastases (BMs) and demonstrated that there were no significant differences in overall survival (OS) and neurological survival (NS) between treatment with SRS and WBRT for BMs. A similar systematic review and meta-analysis by Gaebe et al., [85] also provides evidence that SRS may achieve survival outcomes compared with WBRT in patients with small cell lung cancer, suggesting that some of these patients may benefit from first-line SRS. Given this evidence on quality of life and the shorter treatment duration offered by SRS, it is crucial that these options are discussed with patients.

### Dosimetric analysis

A conformity index of 1 reflects an ideal PTV coverage and a decrease from this indicates a decrease in plan quality. There were variations in the CI_p_ and nCI across the studies, and in some cases this evidence was conflicting. In the GK vs DCA study by Chea *et al*., [25]. GK had better CI_p_ and in a separate GK vs SI-VMAT study by Liu et al., SI-VMAT had better nCI. El Shafie *et al*., [52] and Kadoya *et al*., [26] also reported opposing findings on the CI_p_ for CK vs VMAT based SRS. El Shafie *et al*., [52] used Multiplan (for CK) and RayStationV8 (for SI-VMAT) TPSs and found CK CI_p_, 0.83 (nCI = 1.2) to be more conformal than SI-VMAT SRS which had CI_p_ of 0.63 (nCI = 1.6). On the other hand, Kadoya *et al*., [26] used Multiplan (CK) and Eclipse (for SI-VMAT) TPSs and found CK CI_p_ of 0.60 and less conformal to HyperArc SRS CI_p_ of 0.87. The low CI_p_ for SI-VMAT could be linked to the limitation or ability to plan VMAT SRS by RayStationV8 as also demonstrated by limited publications involving this TPS. The lower CI_p_ on Multiplan (CK) was attributed to the number of tumours by the authors. According to Kadoya *et al*., [26] the lower CI_p_ could be explained from the limitation of cone delivery system on CK, which may not perfectly fit the target especially for multiple targets compared to HyperArc that uses MLCs which can conform to the target [26]. This argument can be easily counteracted since CK can deliver dose from many angles thereby conforming to the target. This demonstrates the complexity of SRS and that the factors influencing the plan quality are not only the complex SRS delivery system.

In a study comparing CK, HyperArc and a standard VMAT SRS, Slosarek *et al*., [86] found minor differences in CI_p_ between the three techniques i.e CK (0.87), HyperArc (0.86) and 0.81 for standard VMAT. In a separate study, Zhang *et al*., [87] assessed plan quality indices for CK, IMRT and coplanar VMAT and non-coplanar VMAT (standard VMAT). They found standard VMAT to have a slightly better CI_p_ (0.9) than CK (0.86). By design and ability to treat from several beam angles it is expected that CK will have a better conformity than VMAT based SRS. However, a potentially significant influence of the planners’ ability and bias on plan quality indices has been suggested by other studies [53].

In general, C-arm linac-based SRS showed CI_p_ and nCI were closer to 1 demonstrating comparable conformality across the techniques. HyperArc consistently performed better than any C-arm linac-based SRS it was compared with except DCA VMAT which marginally performed better (1.25 vs 1.24). The above discussion also highlighted the influence of the planner’s ability in these studies.

CI_RTOG_ was only recorded for studies involving CK. In all cases, the CK had better conformity than the C-arm linac-based SRS. Zhang *et al*., [28] demonstrated that SI-VMAT SRS was more comparable to CK conformality than C-VMAT and IMRT SRS. The ability to deliver non-coplanar multiple beams might be the reason for the SI-VMAT SRS performing slightly better than the latter techniques.

A GI of 3 or less is considered achievable and reasonable [33, 56, 70]. The mean and median GI_p_ were 1.73 and 1.71 higher than the expected Gradient index. This reflects a general difficulty in achieving the ideal gradient index across the techniques. The VMAT vs DCA study by Turkkan *et al*., [64] had the largest GI_p_ difference and yet it’s using the same TPS and algorithm. This could be attributed to the TPS not being fully optimised for that SRS technique. This view is supported by the conflicting findings in DCA vs SI-VMAT. Velten *et al*., [53] found DCA to have better GI_p_. SI-VMAT 6 vs 10FFF and VMAT opt vs VMAT non-opt showed similar GI_p_. Chea *et al*., [25] found the DCA to have better GI_p_ (3.22) compared to GK (4.09). Overall, more studies demonstrated that GK and CK had better dose fall off than C-arm linac-based SRS [26–28, 52]. The sharp dose fall off in GK and CK can be attributed to the ability to deliver dose from many optimised non-coplanar beam angles.

Other studies reported brain V2Gy up to V16Gy, but only brain V12Gy was reported in Table 2 and Fig 6 as many studies consider brain V12Gy < 10cm^3^ to be a technically achievable planning objective with SRS, and a parameter where the risk of radiation necrosis has been collected and reported across various studies [24, 27, 88–91]. Fractionated SRS (SRT) is recommended for brain V12Gy >10 cm^3^ to reduce the risk of radionecrosis [82]. A shortcoming is that some of these studies have often included the GTV tumour in the “brain” volume, when in fact the larger the PTV margin, the more normal brain is treated to high dose. Because some studies report on GK with no margin, others who do add a PTV margin treat normal brain to a higher dose and so we have different rates of radionecrosis reported for the same planning objective in different studies.

A combination of the direct beam and out-of-field (OOF) dose impacts the brain V12Gy. The OOF dose comes from head leakage, collimator, and patient scatter. In addition, the V12Gy depends on the number or and size of tumours. The CK vs SI-VMAT study by Kadoya *et al*., [26] showed CK V12Gy to be approximately 6 times better than SI-VMAT. This huge difference was not observed in any other study and can’t be fully explained. Pokhrel *et al*., [71] found HyperArc to have more V12Gy than DCA-VMAT. Otherwise, the other studies showed comparable V12Gy.

GK and CK demand longer treatment times in comparison to C-arm linac-based SRS. This substantially affects efficiency and serves as a motivating factor to explore other techniques. CK mostly use cones and technically require more MUs. There were also MU variations among the C-arm linac-based SRS. MI-VMAT technique required 3 times MUs to deliver the same dose compared to the HyperArc technique. Furthermore, the VMAT or DCA uses dynamic MLCs and that reduces treatment time. The reviewed papers did not address the time taken per patient from CT simulation to treatment. This time depends on many facets especially the clinical workflows of individual departments and is very important for bench marking especially for centres wanting to start an SRS program or those considering quality improvement.

### Geometric shifts analysis

Studies show that intra-fraction motion for SRS can be up to 1.5mm and 2° [32], therefore the impact on dose coverage is normally studied up to 2mm translational and 2° rational shifts [13]. The compromise in CI_p_ due to translational shifts was similar for both multiple isocentres and single-isocentre. However, the change in CI_p_ was different between multiple-isocentre and single-isocentre for rational shifts. Single-isocentre treatments were greatly impacted compared to multiple isocentre treatments and this was observed by several authors [49, 92, 93]. Selvan *et al*., [94] observed a decrease in PTV coverage for 1–5° rotational error as the tumour radial distance increases from the isocentre in a single-isocentre SRS technique. Nakano *et al*., [49] performed extensive simulation of setup errors involving 6DoF couch and found that target coverage is more compromised by rotational setup errors in single-isocentre treatments. SRS delivery systems that cannot provide set-up accuracy within 0.5mm and 0.5° should not be used for targets that are smaller than 1.5cm in diameter. The study also concluded that there was increased risk of missing the target with increase in separation distance and a decrease in GTV size. This poses a potential risk for poor local control in C-arm linac based single isocentre MBM SRS compared to GK and CK. Although the C-arm linac treatment plan may appear optimal, treatment delivery might be less accurate for distal metastases. Additionally, C-arm linac MBM SRS techniques tend to use larger PTV margins which increases the risk of radionecrosis. C-arm linacs are more widely available compared to GK and CK, which are dedicated SRS systems. The dosimetric and geometric accuracies discussed are comparable and there is a huge benefit from shorter treatment times and more throughput with C-arm linac based SRS.

## Limitations

Most of the treatment plans generated for comparison were “in silico” and no patient specific QA measurements were performed to compare with the plans. Some of the studies did not indicate if PTV margin was the same for different techniques in the same study and hence the same PTV margin per study was assumed. The planners across all the studies may have varied levels of experience and skills, contributing to planner subjectivity in the findings. There is scarcity of reports or studies on overall survival for C-arm linac based MBM SRS as monotherapy. Conducting a randomized trial to investigate overall survival for MBM patients treated with C-arm linac-based single-isocentre SRS compared to GK or CK would be valuable. Some studies have compared patient setup, pre-treatment and during-treatment imaging as well as the actual beam-on time across two or three techniques. However, it would be valuable to conduct studies that include the required time for the whole process from CT simulation to treatment.

## Conclusion

A review of SRS treatment techniques over the past 8 years demonstrated that C-arm linac-based SRS is gaining more traction due to improved planning and delivery mechanisms. There is a growing number of studies comparing single-isocentre SRS technique with traditional SRS techniques. These studies are showing comparable conformity indices between GK, CK, and C-arm linac-based SRS techniques. Overall GK and CK showed slightly better gradient index and sparing of normal brain. However, in some cases, HyperArc and DCA have better sparing of normal brain, especially in cases with multiple targets. GK and CK SRS treatments take much longer to deliver compared to C-arm linac-based SRS. Rotational errors are a major source of dosimetric error in single-isocentre SRS techniques and 6DoF geometric corrections are highly recommend. Care should be taken for large PTV to isocentre distance as it increases the healthy brain dose. As centres with C-arm linac based SRS gain confidence with their techniques, there is potential for reducing PTV margins, thereby reducing the risk of radionecrosis. Developing software capable of real time calculation of geometric accuracy real time calculation during MBM SRS delivery would be beneficial. Overall, the maturity of C-arm linac-based SRS has been demonstrated and should be rolled out in departments with capable linac-based facilities.

## Supporting information

S1 Checklist (PDF)

S2 Checklist PRISMA 2020 checklist. (DOCX)

S1 Data (XLSX)
