# Supplementary material for: Insights into the dosimetric and geometric characteristics of stereotactic radiosurgery for multiple brain metastases: A systematic review
Source: PLoS One. 2024 Aug 9;19(8):e0307088. doi: 10.1371/journal.pone.0307088 (PMC11315342; doi:10.1371/journal.pone.0307088)
Supplement: S1 Checklist — (PDF) [file pone.0307088.s001.pdf]

CASP
Critical Appraisal
Skills Programme

CASP Checklist: 10 questions to help you make sense of a **Systematic Review**

How to use this appraisal tool: Three broad issues need to be considered when appraising a systematic review study:

- Are the results of the study valid? (Section A)
- What are the results? (Section B)
- Will the results help locally? (Section C)

The 10 questions on the following pages are designed to help you think about these issues systematically. The first two questions are screening questions and can be answered quickly. If the answer to both is "yes", it is worth proceeding with the remaining questions. There is some degree of overlap between the questions, you are asked to record a "yes", "no" or "can't tell" to most of the questions. A number of italicised prompts are given after each question. These are designed to remind you why the question is important. Record your reasons for your answers in the spaces provided.

About: These checklists were designed to be used as educational pedagogic tools, as part of a workshop setting, therefore we do not suggest a scoring system. The core CASP checklists (randomised controlled trial & systematic review) were based on JAMA 'Users' guides to the medical literature 1994 (adapted from Guyatt GH, Sackett DL, and Cook DJ), and piloted with health care practitioners.

For each new checklist, a group of experts were assembled to develop and pilot the checklist and the workshop format with which it would be used. Over the years overall adjustments have been made to the format, but a recent survey of checklist users reiterated that the basic format continues to be useful and appropriate.

Referencing: we recommend using the Harvard style citation, i.e.: *Critical Appraisal Skills Programme (2018). CASP (insert name of checklist i.e. Systematic Review) Checklist. [online] Available at: URL. Accessed: Date Accessed.*

©CASP this work is licensed under the Creative Commons Attribution – Non-Commercial-Share A like. To view a copy of this license, visit http://creativecommons.org/licenses/by-nc-sa/3.0/ www.casp-uk.net

CASP
Critical Appraisal Skills Programme

Paper for appraisal and reference:

Section A: Are the results of the review valid?

1. Did the review address a clearly focused question?

Yes ✔
Can't Tell ☐
No ☐

HINT: An issue can be 'focused' In terms of
- the population studied
- the intervention given
- the outcome considered

Comments: The review focused on multiple brain metastases (MBM) stereotactic radiosurgery (SRS) for a specific period.

2. Did the authors look for the right type of papers?

Yes ✔
Can't Tell ☐
No ☐

HINT: 'The best sort of studies' would
- address the review's question
- have an appropriate study design (usually RCTs for papers evaluating interventions)

Comments: The reviewed papers covered a wide range of MBM SRS techniques.

Is it worth continuing?

3. Do you think all the important, relevant studies were included?

Yes ✔
Can't Tell ☐
No ☐

HINT: Look for
- which bibliographic databases were used
- follow up from reference lists
- personal contact with experts
- unpublished as well as published studies
- non-English language studies

Comments: At least three major databases (Scopus, Pub Med, Web of science and other relevant sources were interogated.

4. Did the review's authors do enough to assess quality of the included studies?

Yes ☑
Can't Tell ☐
No ☐

HINT: The authors need to consider the rigour of the studies they have identified. Lack of rigour may affect the studies' results ("All that glisters is not gold" Merchant of Venice – Act II Scene 7)

Comments: Two independent authors assessed the quality of the remaining 20 articles.

5. If the results of the review have been combined, was it reasonable to do so?

Yes ☑
Can't Tell ☐
No ☐

HINT: Consider whether
- results were similar from study to study
- results of all the included studies are clearly displayed
- results of different studies are similar
- reasons for any variations in results are discussed

Comments: Similar plan qualities were compared and discussed.

Section B: What are the results?

6. What are the overall results of the review?

HINT: Consider
- If you are clear about the review's 'bottom line' results
- what these are (numerically if appropriate)
- how were the results expressed (NNT, odds ratio etc.)

Comments: C-arm linac-based SRS produce similar plan qualities to the mainstay SRS techniques.

CASP
Critical Appraisal
Skills Programme

7. How precise are the results?

HINT: Look at the confidence intervals, if given

Comments: No statistical analysis.

Section C: Will the results help locally?

8. Can the results be applied to the local population?

Yes ✔
Can't Tell ☐
No ☐

HINT: Consider whether
- the patients covered by the review could be sufficiently different to your population to cause concern
- your local setting is likely to differ much from that of the review

Comments: Yes, plan qualities are independent of geographical settings, etc.

9. Were all important outcomes considered?

Yes ✔
Can't Tell ☐
No ☐

HINT: Consider whether
- there is other information you would like to have seen

Comments: Yes, all relevant outcomes were reported & discussed.

10. Are the benefits worth the harms and costs?

Yes ✔
Can't Tell ☐
No ☐

HINT: Consider
- even if this is not addressed by the review, what do **you** think?

Comments: Yes, more access to SRS techniques which are good for reducing radiation dose to the patients.
